# Increased Expression of Bacterial Cellulose Synthase Genes in *Komagataeibacter Xylinus* Exposed to a Rotating Magnetic Field

**DOI:** 10.1021/acs.biomac.5c00653

**Published:** 2025-08-29

**Authors:** Anna Zywicka, Aleksandra Dunisławska, Karol Fijalkowski

**Affiliations:** † Department of Microbiology and Biotechnology, West Pomeranian University of Technology in Szczecin, Piastow 45, Szczecin 70-311, Poland; ‡ Department of Animal Biotechnology and Genetics, 111405 Bydgoszcz University of Science and Technology, Mazowiecka 28, Bydgoszcz 85-084, Poland

## Abstract

The objective of this study was to investigate the alterations in the expression of *bacterial cellulose synthase* (*bcs*) genes in*Komagataeibacter xylinus*, dependent on the exposure duration and specific parameters of a rotating magnetic field (RMF).*K. xylinus* cells were subjected to an RMF at frequencies of 5 and 50 Hz for durations ranging from 12 to 72 h. Gene expression was assessed using quantitative polymerase chain reaction (qPCR). The findings demonstrated that RMF exposure significantly enhanced BC production efficiency. Specifically, the average yields of wet and dry BC following RMF treatment increased by 28% and 18%, respectively, compared to the control. These improvements correlated with upregulated *bcs* gene expression, which showed statistically significant changes at both frequencies and all time points. The strongest effects occurred at 5 Hz. In conclusion, RMFs represent a promising approach to modulate gene expression in bacteria, offering substantial biotechnological potential, particularly for enhancing BC production.

## Graphical abstract

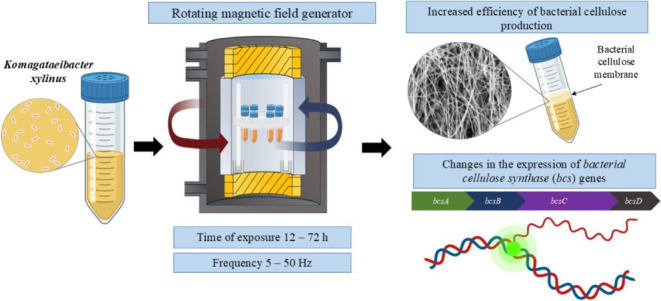

## Introduction

Acetic acid bacteria of the genus *Komagataeibacter* are exceptionally proficient producers of bacterial cellulose (BC).^1^ Specifically, *Komagataeibacter xylinus* (formerly *Gluconacetobacter xylinus*) is frequently employed as a model microorganism in studies concerning the biochemistry and genetics of BC biosynthesis.^2^ Structurally, BC is a simple polysaccharide composed of glucose units, which are linearly arranged and linked by β-1,4 glycosidic bonds. Compared to plant-derived cellulose, BC exhibits superior properties, including high tensile strength, crystallinity, porosity, water-swelling capacity, and biocompatibility.^3^ These unique characteristics underpin BC’s extensive applications in both medical and industrial sectors, such as in wound dressings for patients with severe burns and injuries, as carriers in drug delivery systems, implants, contact lenses, tympanic membranes, and artificial corneas.^3–5^ In the industrial domain, BC is utilized in the production of high-quality paper, as a carrier for cell immobilization, as a food additive, and as a filtration material for air and water purification.^4–7^ Despite its promising potential, the high production cost of BC remains a significant barrier, limiting its adoption in both the industrial and medical fields compared to its vast application possibilities.

Bacterial cellulose biosynthesis in *K. xylinus* is regulated by the bcs operon, which encodes the proteins comprising the BC synthesis complex. This operon consists of four genes—*bcsA, bcsB, bcsC,* and *bcsD*—each with distinct roles in bacterial cellulose biosynthesis.^8,9^ The gene *bcsA* encodes the catalytic subunit of cellulose synthase, which is responsible for polymerizing uridine diphosphoglucose (UDP-glucose) monomers into β-1,4-glucan chains, forming cellulose.^10^ The product of the *bcsB* gene serves as the regulatory subunit of cellulose synthase. It binds to cyclic di-GMP, a critical second messenger that activates the cellulose synthesis process.^9,10^ While the function of *bcsC* is not fully elucidated, this gene is believed to facilitate the formation of pores in the outer cell membrane and participate in the export of the synthesized polysaccharide to the extracellular space. The *bcsD* gene’s product is hypothesized to be involved in the crystallization of cellulose into nanofibrils, although its precise function remains to be fully characterized. It is also worth noting that the roles of *bcsC* and *bcsD* remain subject to ongoing research and debate within the scientific community.^11,12^

Since the early 1970s, numerous studies have demonstrated that magnetic fields (MFs) can affect various functional parameters of microorganisms, including the growth rate, metabolic activity, biofilm formation, and viability. The influence of MFs on these bacterial functional parameters is contingent upon several factors, such as MF intensity, duration of exposure, and the specific type of organism exposed.^13,14^ Several studies also highlight the direct and indirect effects of MFs on the DNA structure and gene expression in living organisms. For instance, it has been observed that MFs can induce DNA degradation in *E. coli*.^15^ In *Salmonella enterica* subsp. *enterica*, MF exposure has been shown to elevate the expression of the *katN* gene, which encodes a non-heme catalase, the *dnaK* gene, which encodes a heat shock protein, and the *rpoA* gene, responsible for the α-subunit of RNA polymerase.^16^ Moreover, MF exposure influences the *relA* and *spoT* genes, crucial for the synthesis and degradation of *(p)­ppGpp* (guanosine pentaphosphate or tetraphosphate), a pleiotropic regulator involved in the stringent response and other stress responses in bacteria.^17^ Mouhoub et al.^18^ further confirmed the impact of MFs on the expression of genes involved in the biosynthesis of cardiolipin, such as *g3pd, pgsA,* and *cls*, as well as phosphatidylethanolamine biosynthesis genes *pssA* and *psd* in *Salmonella enterica* subsp. *enterica*. Similarly, our study demonstrated that MF exposure significantly affects the expression of genes encoding staphylococcal enterotoxins, including *sea, sec,* and *sel,* ^19^ along with *seb*, *see*, and *sed.* ^20^ Furthermore, various studies suggest that exposure to MFs may induce DNA damage, such as the occurrence of point mutations, as demonstrated by Potenza et al.^21^ Additionally, MFs may contribute to DNA degradation, either through direct interactions with the molecular structure or via the generation of reactive oxygen species.^15^

In our investigation into the potential application of MFs in BC production, we observed that exposure to a rotating magnetic field (RMF) markedly promotes the growth and metabolic activity of *K. xylinus* cells.^22–25^ Specifically, we observed that applying an RMF at a frequency of 50 Hz for 72 h resulted in a substantial increase in BC yield compared with the unexposed control. Additionally, it has been reported that the RMF promotes the production of a *K. xylinus* inoculum with a cellular density 200 times higher, maintaining high and stable metabolic activity throughout the BC biosynthesis process.^26^ Given our previous findings, it can be inferred that this innovative approach, leveraging the RMF, has the potential to enhance the efficiency of BC production. However, to fully control the BC production process, it is essential to investigate the molecular and cellular mechanisms underlying the stimulating effect of the RMF on *K. xylinus* cells.^12^

Building upon our previous findings related to the influence of MFs on bacterial gene expression, we hypothesized that the enhanced efficiency of BC production observed in *K. xylinus* exposed to the RMF may be partially linked to increased expression of genes encoding the multicomponent protein complex known as *bacterial cellulose synthase* (bcs). This complex is crucial for regulating key processes involved in the biosynthesis of BC. Consequently, the objective of this study was to investigate the effects of RMFs on the expression of the *bcsA, bcsB, bcsC,* and *bcsD* genes in *K. xylinus*, taking into account different exposure parameters and durations.

## Materials and Methods

### Rotating Magnetic Field Generator

The experiments were conducted utilizing a self-designed RMF exposure system specifically adapted for biological studies, as illustrated in Figure 1 and detailed in our previous publications.^19,22^ The RMF generator was constructed based on a three-phase, four-pole stator with an internal diameter of 16 cm and a height of 20 cm, comprising 12 groups of three coil sets. The frequency of the alternating current (AC) supplied to the RMF generator was regulated using a Unidrive M200 inverter (Control Techniques, Nidec Industrial Automation, Poznań, Poland). Temperature control within the RMF reactor chamber was achieved via a water thermostat (KISS K6, Huber, Germany), equipped with a series of temperature sensors with an accuracy range of ±1.0 °C. A consistent temperature distribution in the RMF bioreactor was maintained with an airflow rate of 2 L/min at 35 °C and 60% relative humidity. The distribution of magnetic induction (*B*) in the process chamber was determined at 100 V and AC frequencies of 5 and 50 Hz using the Ansys Maxwell simulation software ver.19.1 (ANSYS Inc., USA) and confirmed empirically using a teslameter (SMS-102, Asonik, Tuczno, Poland) equipped with a transverse probe. A detailed description of the RMF generator parameters and a graphical presentation of the arrangement of the test tubes during the exposure to the RMF are provided in Tables S1 and S2 and Figure S1.

**Figure 1:**
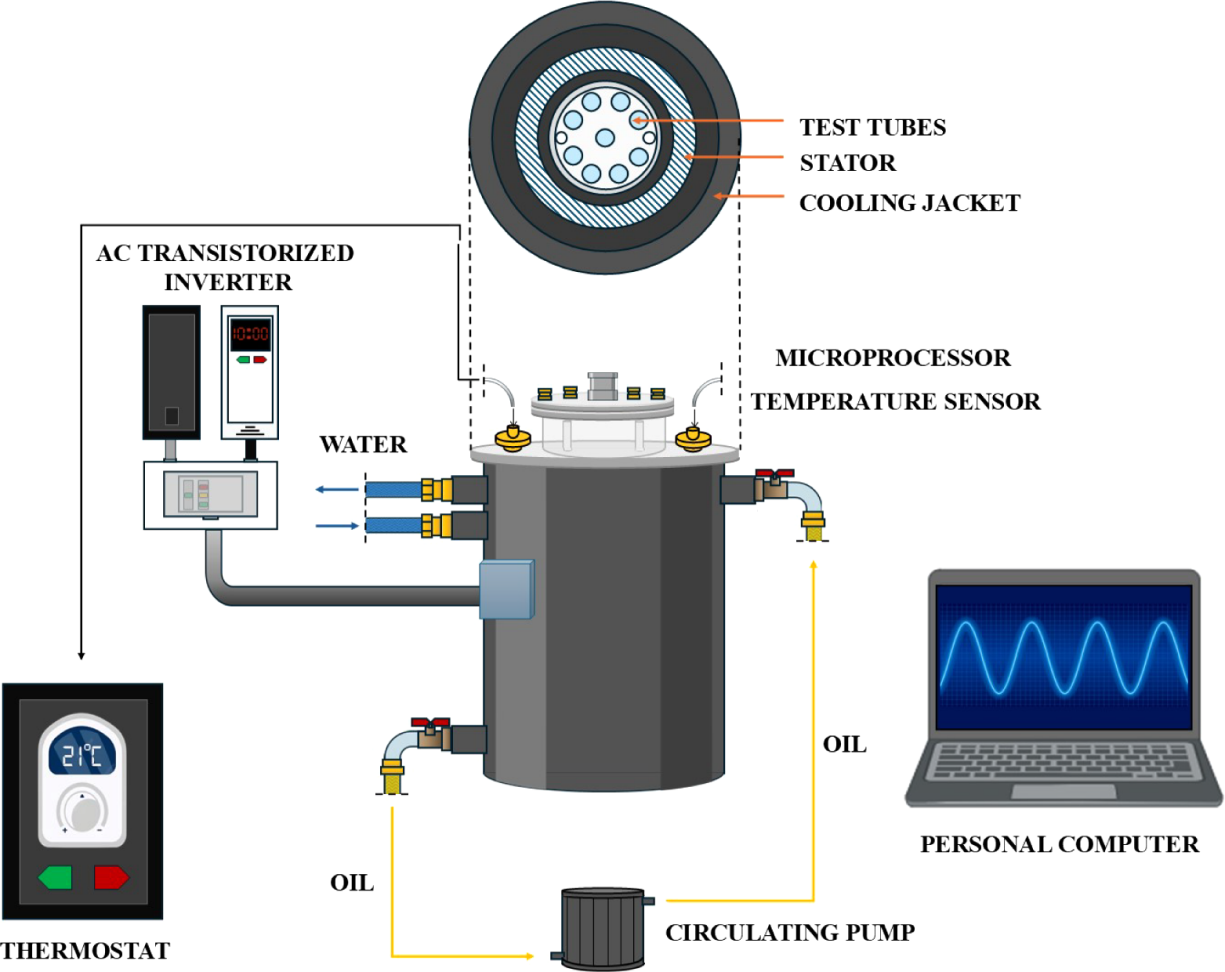
RMF generator with monitoring and control equipment. This figure was created by using BioRender.com.

### Exposure of *K. xylinus* to a Rotating Magnetic Field

Prior to each experiment, the reference strain of *Komagataeibacter xylinus* (American Type Culture Collection [ATCC] 53582) was cultivated under stationary conditions using a Hestrin–Schramm (HS) medium composed of glucose (2% w/v), yeast extract (0.5% w/v), bacto-peptone (0.5% w/v), citric acid (0.115% w/v), Na_2_HPO_4_ (0.27% w/v), MgSO_4_·7H_2_O (0.05% w/v), bacteriological agar (2% w/v), and ethanol (1% v/v) for a duration of 7 days at 28 °C. Following this incubation period, the 7-day culture was vigorously agitated to detach cells from the cellulose membrane. A bacterial suspension with a cell density of 2 × 10^6^ CFU/mL was then used to inoculate 25 mL of medium in 50 mL plastic tubes (with a diameter of 3.8 cm) containing caps with eight holes and a specialized capillary pore filter membrane (CELLSTAR® CELLreactor, Polypropylene Filter Top Tube, Greiner Bio-One, USA).

The *K. xylinus* cultures were subsequently exposed to the RMF generator for 12, 24, 48, or 72 h at an incubation temperature of 28 °C, during which two different frequencies, 5 Hz (the minimum frequency achievable by the RMF generator) and 50 Hz (the maximum frequency achievable by the RMF generator), were applied. For control purposes, *K. xylinus* cultures were cultivated for the same duration and under identical conditions without exposure to the RMF. The arrangement and positioning of the test tubes within the RMF reactor chamber are illustrated in Figure 2.

**Figure 2:**
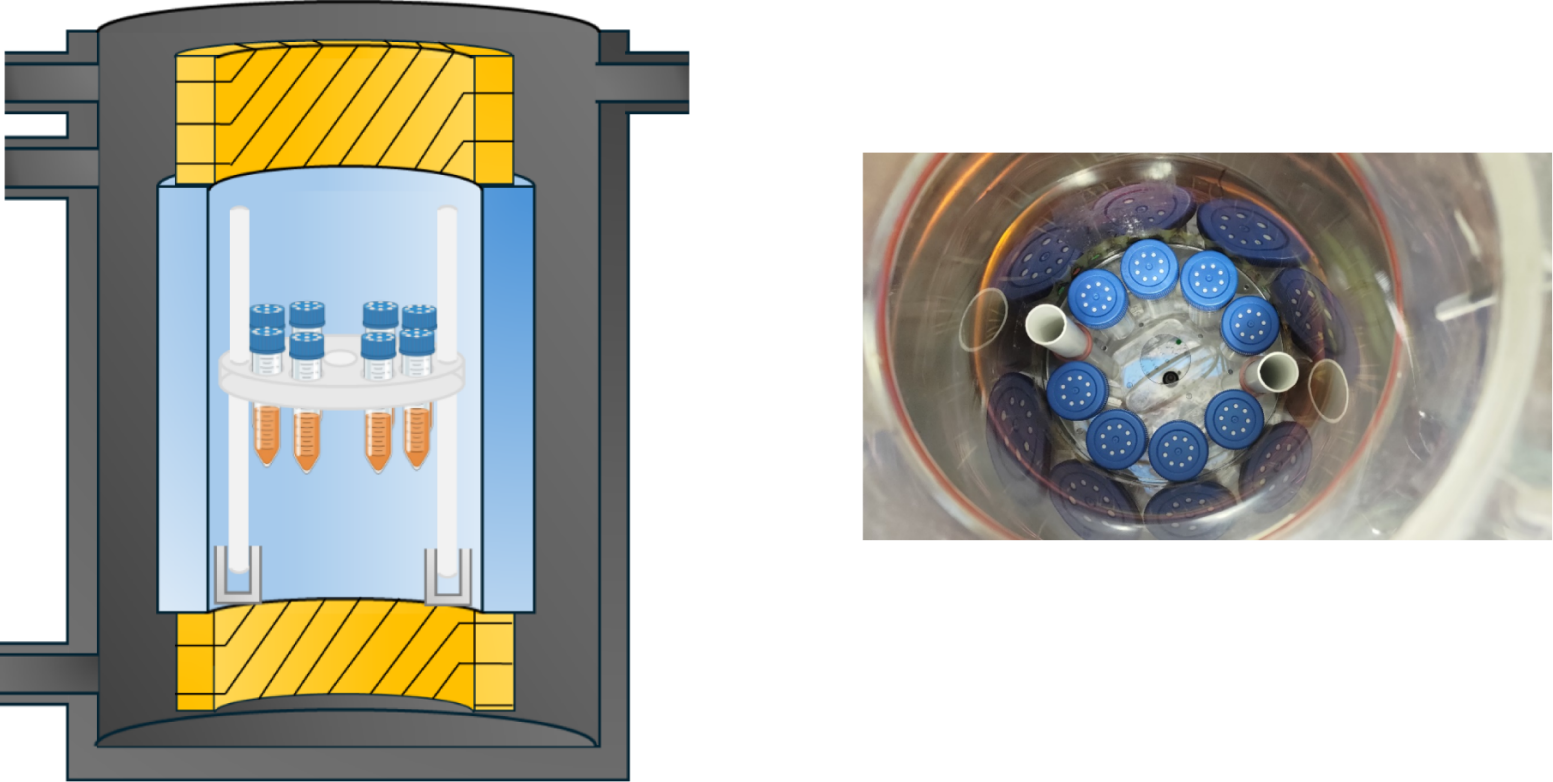
Arrangement and location of the test tubes in the RMF reactor chamber. The left part of this figure was created using BioRender.com.

### Quantification and Viability Assessment of *K. xylinus* Cells

The quantity and viability of *K. xylinus* cells were assessed over a 72 h incubation period using two distinct approaches. First, cell viability in the liquid HS medium was evaluated after removing the cellulose pellicle from the culture tube (referred to as “cells in medium”). Second, the viability of cells embedded within the cellulose pellicle (referred to as “cells in cellulose”) was determined following enzymatic digestion of the pellicle using cellulase. Both assessments employed the alamarBlue Cell Viability Assay (Thermo Fisher, USA), a ready-to-use, resazurin-based reagent that serves as an indicator of cellular metabolic activity and viability.^27^

For enzymatic digestion, cellulose pellicles were first washed with distilled water and then transferred into 25 mL of a cellulase solution prepared in 0.05 mol/L citrate buffer (pH 4.8). The samples were incubated while being shaken at 30 °C for 6 h. Following digestion, both the culture medium and the resulting cell suspensions from pellicle hydrolysis were centrifuged at 3300 × *g* for 10 min. The resulting pellets were washed once with phosphate-buffered saline (PBS; MilliporeSigma, USA), centrifuged again under the same conditions, and resuspended to their original volume with PBS. Aliquots of the bacterial suspensions (200 μL) were transferred into 96-well fluorescence microtiter plates (Becton Dickinson and Company, USA), followed by the addition of 20 μL of alamarBlue reagent. The plates were incubated in the dark at 30 °C for 1 h. Fluorescence was measured using a microplate reader (Synergy HTX, BioTek, USA) with excitation and emission wavelengths of 540 and 590 nm, respectively. Sterile PBS served as the blank control.

### Evaluation of Bacterial Cellulose Yields

#### To Evaluate the BC Yield, the Samples Were Subjected to a Standardized Two-Phase Process

- RMF exposure: samples were exposed to the RMF under conditions specified in the preceding chapter.
- Post-RMF incubation: following RMF exposure, samples were incubated at 28 °C without exposure to a magnetic field.

The total duration of the experiment, encompassing both RMF exposure and subsequent incubation, was maintained at 72 h for all samples. This standardized approach ensured uniformity under experimental conditions across all specimens, allowing for accurate assessment of the BC yield under varying RMF exposure times.

After 72 h of incubation, the BC membranes were harvested and purified by treating them with a 0.1 M NaOH solution at 80 °C for 90 min. Next, the samples were rinsed with distilled water until the pH reached neutrality (pH 6.5–7.5) and then weighed on an analytical balance (with an accuracy of 0.0001 g; WTB 2000 Radwag, Radom, Poland). Subsequently, the BC membranes were dried at 60 °C for 12 h and weighed again. The yield of BC was expressed as either wet or dry weight (in grams) per 100 mL of the culture medium.

### Assessment of the Structural Properties of BC Modified with the RMF

The BC microstructure was assessed using scanning electron microscopy (SEM, Auriga 60, Zeiss, Jena, Germany).^24^ The chemical composition of the BC membranes was determined by using attenuated total reflectance-Fourier transform infrared spectroscopy (ATR-FTIR, ALPHA FTIR spectrometer, Bruker Co., Leipzig, Germany). The total crystallinity index (TCI), lateral order index (LOI), and hydrogen bond index (HBI) of BC samples were determined as the ratio of absorbance values for bands 1370 cm^–1^ and 2900 cm^–1^, 897 cm^–1^ and 1429 cm^–1^, and 1320 cm^–1^ and 3400 cm^–1^, respectively.^26^ To examine the water-related properties of BC, the cellulose pellicles were dried at 60 °C for 6 h to remove any water content and weighed again. In the next step, the samples were immersed in distilled water until maximum absorption was reached and weighed again. They were then wiped carefully with filter paper, placed on the analytical balance, weighed, incubated for 60 h at 25 °C, and weighed again.^28^ The weights of the BC samples were taken in triplicate.

The water-holding capacity (%WHC) was calculated by the formula:

$$\mathrm{\%WHC}=\frac{\left(W_{\mathrm{wet}}-W_{\mathrm{dwet}}\right)}{W_{\mathrm{dry}}}\times 100\%$$

Where *W*_wet_ is the weight of the swollen BC, *W*_dwet_ is the weight of the swollen BC during drying, and *W*_dry_ is the weight of the dry sample.

The swelling ratio (SR%) was calculated by the formula:

$$\%SR=\frac{\left(W_{wet}-W_{dry}\right)}{W_{dry}}\times 100\%$$

where, *W*_wet_ is the weight of the swollen BC, and *W*_dry_ is the weight of the dry sample.

### Evaluation of Gene Expression

#### RNA Extraction

RNA extraction was performed after 12, 24, 48, and 72 h of RMF exposure. The whole *K. xylinus* culture (medium and cellulose pellicle) was vigorously shaken to detach cells from the cellulose membrane. The cellulose pellicle was removed, and the medium containing the released cells was centrifuged for 10 min at 1500 × *g*. The bacterial suspension, with a cell density of approximately 2 × 10^8^ CFU/mL in PBS, was transferred to 1.5 mL tubes and centrifuged again. Then, the PBS was replaced with 1 mL of RNA protection reagent (stayRNA, A&A Biotechnology, Poland), and the cells were frozen at −82 °C until RNA extraction.

The next day, samples were thawed to room temperature, and total RNA was isolated using the RNA Isolation Mini Kit (A&A Biotechnology, Poland) according to the manufacturer’s instructions. RNase-free DNase I (A&A Biotechnology, Poland) was utilized to eliminate any potential DNA contamination. The total RNA concentration was quantified using Qubit RNA Assay Kits (Thermo Scientific, Wilmington, USA), while purity was assessed spectrophotometrically (A_260_ nm/A_280_ nm) using a microplate reader (Synergy HTX, Biotek, USA). The RNA samples were stored at −82 °C until further analysis.

#### Reverse-Transcription and Quantitative Polymerase Chain Reaction

Isolated RNA was reverse-transcribed to complementary DNA (cDNA) using the TranScriba Kit (A&A Biotechnology, Poland) in accordance with the manufacturer’s protocol. Following this, quantitative polymerase chain reaction (qPCR) was performed utilizing a reaction mixture comprising 5 μL of SYBR Green qPCR Master Mix (A&A Biotechnology, Gdańsk, Poland), 1 μg of cDNA, and 1 μM each of forward and reverse primers. qPCR primers were designed and validated for four genes involved in *K. xylinus* BC production (bcsA, bcsB, bcsC, and bcsD; Table 1), and also two reference genes (*23SrRN*A and *gyrB*; Table 1).^8^ Housekeeping genes were chosen based on gene stability analysis using the RefFinder tool (Table S3 and Figures S2–S4).

**Table 1:** Primer Sequences Used in qPCR Reactions

Gene		Sequence (5’→3’)	Product length (bp)
***23Sr RNA***	Forward	TGAGCTGGGTTTAGAACGTCGTG	255
	Reverse	ACACCTGGCCTATTGACGTGATG	
***gyrB***	Forward	TCTCGTCACAGACCAAGGACAAG	108
	Reverse	CTTCCTTGGGGTGGGTTTCAAAC	
***bcsA***	Forward	ACAATGGGCTGGATGGTCGA	184
	Reverse	ACCCGCAAAAGAAGGTCGCA	
***bcsB***	Forward	AATGCGTTCCATCTTGGGCTTGAC	197
	Reverse	ATCAGGTCAAGATAGGCGCCAACA	
***bcsC***	Forward	TACCAGTCGCATATCGGCAATCGT	103
	Reverse	GCAGGTCGTTCAACTGGCTTTCAT	
***bcsD***	Forward	TCACCCTGTTTCTTCAGACCCTGT	153
	Reverse	TCAGTTCGATCTGCAGCTTGTCCA	

The qPCR reactions were carried out using the LightCycler® 480 Instrument II (Roche Diagnostics, Basel, Switzerland). The thermal cycling program included an initial denaturation step at 95 °C for 20 min, followed by 40 amplification cycles consisting of denaturation (15 s at 95°C), annealing (20 s at 62 °C for *23SrRNA*, *bcsB*, and *bcsD;* 60 °C for *gyrB, bcsA*, and *bcsC*), and elongation (20 s at 72°C). Each qPCR reaction was conducted in duplicate. Gene expression for the bcs genes was analyzed using a modified delta-Ct relative quantification model, which included corrections for PCR efficiency and normalization to the housekeeping genes *23SrRNA* and *gyrB*.^29^ The results are presented as normalized quantities for each *bcs* gene in *K. xylinus* cells exposed to the RMF compared with the unexposed controls.

### Statistical Analysis

All experiments were performed using three independent biological replicates (*n* = 3), unless stated otherwise. For quantitative real-time PCR (qPCR), each biological replicate was analyzed in duplicate (two technical replicates), and the average of the technical replicates was used for statistical analysis. For cellulose yield measurements, each data point represents the mean of three biological replicates. Results are reported as mean ± standard deviation (SD), based on biological replicates (error bars in all figures reflect biological variability). Statistical comparisons between RMF-exposed and unexposed control samples were conducted using one-way analysis of variance (ANOVA), followed by Tukey’s posthoc test for multiple comparisons. Statistical significance was defined as follows: *p* < 0.05 (*), *p* < 0.01 (**), *p* < 0.001 (***), and *p* < 0.0001 (****).

## Results and Discussion

### Quantification and Viability Assessment of *K. Xylinus* Cells

The kinetics of bacterial growth and the dynamics of BC production are critical for determining the optimal duration and conditions for *K. xylinus* cultivation.^30^ Furthermore, it is well established that gene expression in bacteria is influenced by factors such as growth phase, cell density, and strain specificity.^31,32^ In the present study, *K. xylinus* cell viability was assessed over a 72 h incubation period under exposure to the RMF at frequencies of 5 and 50 Hz using the alamarBlue assay. Cultures not exposed to the RMF served as the control group. As shown in Figure 3, the viability of *K. xylinus* cells, both in the HS medium (after 12 h of incubation) and within the cellulose pellicle, was significantly higher in RMF-exposed samples than in the control. This effect was particularly pronounced at 5 Hz. Specifically, after 12 h of incubation, cell viability in HS medium increased by 16% and 11% for 5 and 50 Hz RMF exposure, respectively, relative to unexposed cultures. For cells embedded in the cellulose pellicle, viability increased by 15–20% at 5 Hz and 5–10% at 50 Hz, depending on incubation time.

**Figure 3:**
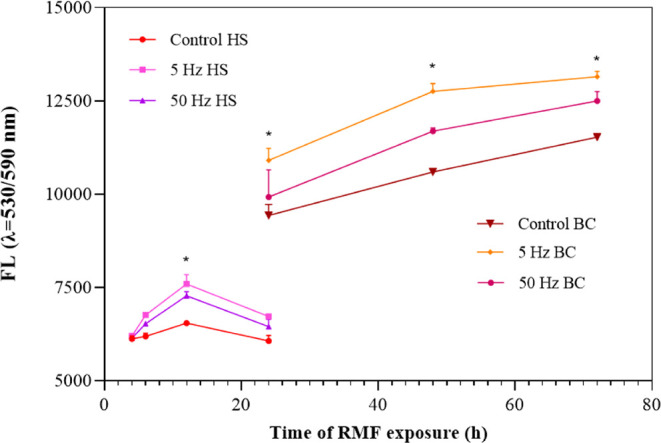
Viability of *K. xylinus* cells in HS medium and within the BC pellicle during 72 h of exposure to the RMF, assessed using fluorescence intensity of the alamarBlue reagent.

These findings align with previous studies, which demonstrated that RMF exposure at 50 Hz for 72–144 h stimulates the growth and metabolic activity of BC-producing *K. xylinus* cells.^22–24^ Additionally, prior research confirmed that RMF exposure, depending on frequency and duration, can modulate growth dynamics, metabolic activity, and biochemical properties of *Staphylococcus aureus* and *Escherichia coli* (RMF frequency: 1–50 Hz; magnetic induction: 22–34 mT).^33,34^ Similar RMF-stimulated effects have been observed in *Serratia marcescens*, *Streptococcus mutans*, *Cronobacter sakazakii*, *Klebsiella oxytoca*, and *Staphylococcus xylosus*.^35^

Data are presented as the mean ± standard error of the mean (SEM). Asterisks (*) indicate statistically significant differences between RMF-exposed samples and unexposed controls (*p* < 0.05).

### Evaluation of the Bacterial Cellulose Yield

In the initial stage of the experiment, we evaluated the yield of BC to validate the preliminary hypotheses formulated based on our previous findings.^19,20^ The results obtained from this study corroborated our assumptions, demonstrating that the RMF had a significant impact on the BC yield compared to the unexposed control. The average yield of BC obtained after RMF exposure (considering all the experiments) was 9.10 g/100 mL and 0.18 g/100 mL for wet and dry BC, respectively, representing increases of 28% and 18% compared to the unexposed control BC (7.10 g/100 mL and 0.16 g/100 mL for wet and dry BC, respectively) (Figure 4).

**Figure 4:**
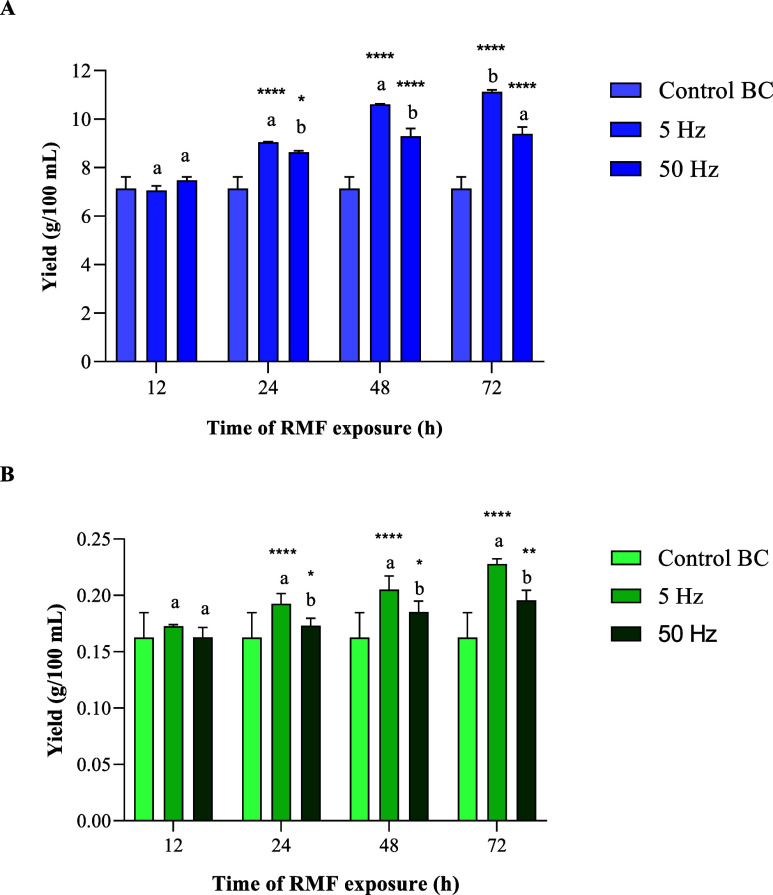
Wet (A) and dry (B) BC yields after exposure to the RMF at different frequencies. The results are presented as the mean ± standard error of the mean (SEM). The “*” symbol indicates statistically significant differences between RMF-exposed samples and unexposed controls (* *p* < 0.05; ** *p* < 0.01; ***p < 0.001; **** *p* < 0.0001). Different letters indicate statistically significant differences between samples exposed to the RMF at different frequencies (Hz) within a specific time point.

The results of the current study also indicated that statistically significant differences in BC yield were dependent on the frequency of the RMF and the time of exposure. The yield of BC obtained after exposure to an RMF of 5 Hz was, on average, 11% higher compared to that obtained at 50 Hz. At both tested frequencies, the BC yields were greater than those of the unexposed controls; however, statistically significant differences were observed when the RMF exposure lasted longer than 12 h. The highest BC yield (11.18 g/100 mL of wet BC) was achieved after 72 h of exposure to the RMF at 5 Hz, which was 56% higher than that of the unexposed controls (Figure 4).

The results obtained in this study are consistent with our earlier observations.^22–26^ We previously proved the positive influence of the RMF on both the production rate and quality parameters of BC synthesized by *K. xylinus*.^22^ This earlier study revealed that BC produced under RMF influence (a frequency of 50 Hz and an exposure duration of 72 h) exhibited higher water absorption, lower density, and fewer interassociated microfibrils compared with the unexposed control. The observed effects were correlated with the duration and timing of magnetic exposure during the cultivation of *K. xylinus*.^23,24^ Additionally, Drozd et al.^25^ confirmed the positive impact of the RMF on cellulose production efficiency, which varied depending on the strain of *K. xylinus* and the frequency used.

#### Assessment of the Structural Properties of BC Modified with the RMF

In our previous study, we demonstrated that *K. xylinus* exposed to the RMF at a frequency of 50 Hz for 72 h produced BC with higher water absorption, lower density, and reduced microfibril interassociation compared to the control.^22^ However, despite these structural differences and the observed impact on biosynthesis efficiency, RMF exposure did not affect the physicochemical properties of BC, as indicated by the crystallinity degree and thermal profile analysis. Similar findings were observed in the current study. The ATR FT-IR spectra of BC from RMF-exposed and control cultures were nearly identical (Figure S5), and the calculated crystallinity indices (LOI, TCI, and HBI), as well as the proportion of the Iα allomorph, showed no statistically significant differences between the two groups (*p* < 0.05). Although BC synthesized under 50 Hz RMF showed slightly higher crystallinity index values compared to those under both the 5 Hz and control conditions, these differences were not statistically significant (Table 2). SEM analysis revealed that BC produced under RMF conditions exhibited a looser nanofibrillar network (Figure S6), which correlated with enhanced water-related parameters relative to the control (Table 2).

**Table 2:** Selected Properties of Unmodified and RMF-Modified BC^a^

	TCI	LOI	HBI	Iα	SR%	WHC%
Control	1.224 ± 0.101^a^	1.137 ± 0.157^a^	0.718 ± 0.042^a^	0.738 ± 0.025^a^	416 ± 73^a^	17 849 ± 245^a^
5 Hz	1.295 ± 0.051^a^	1.192 ± 0.178^a^	0.768 ± 0.064^a^	0.786 ± 0.010^a^	560 ± 124^b^	19 885 ± 342^b^
50 Hz	1.359 ± 0.061^a^	1.283 ± 0.136^a^	0.937 ± 0.045^a^	0.842 ± 0.020^a^	669 ± 178^b^	21 563 ± 398^b^

a TCI is the crystallinity index derived from the absorbance ratio between the wavenumbers 1370 cm^–1^ and 2900 cm^–1^.LOI is the crystallinity index derived from the absorbance ratio between the wavenumbers 897 cm^–1^ and 1429 cm^–1^.HBI is the crystallinity index derived from the absorbance ratio between the wavenumbers 1320 cm^–1^ and 3400 cm^–1^.

### Evaluation of *Bacterial Cellulose Synthase* (*bcs*) Gene Expression

In this study, we investigated the impact of RMF exposure on the expression of these four *bcs* genes in *K. xylinus*. Our findings, as illustrated in Figure 5, reveal that exposure to the RMF significantly modulated the expression of *bcs* genes compared to unexposed control samples. The expression levels of *bcs* genes in *K. xylinus* were found to be influenced by the frequency of the RMF. Compared to the unexposed controls, cells subjected to 5 and 50 Hz RMF exhibited statistically significant differences in *bcs* gene expression. This observation was consistent across all time points and for each of the analyzed genes.

**Figure 5:**
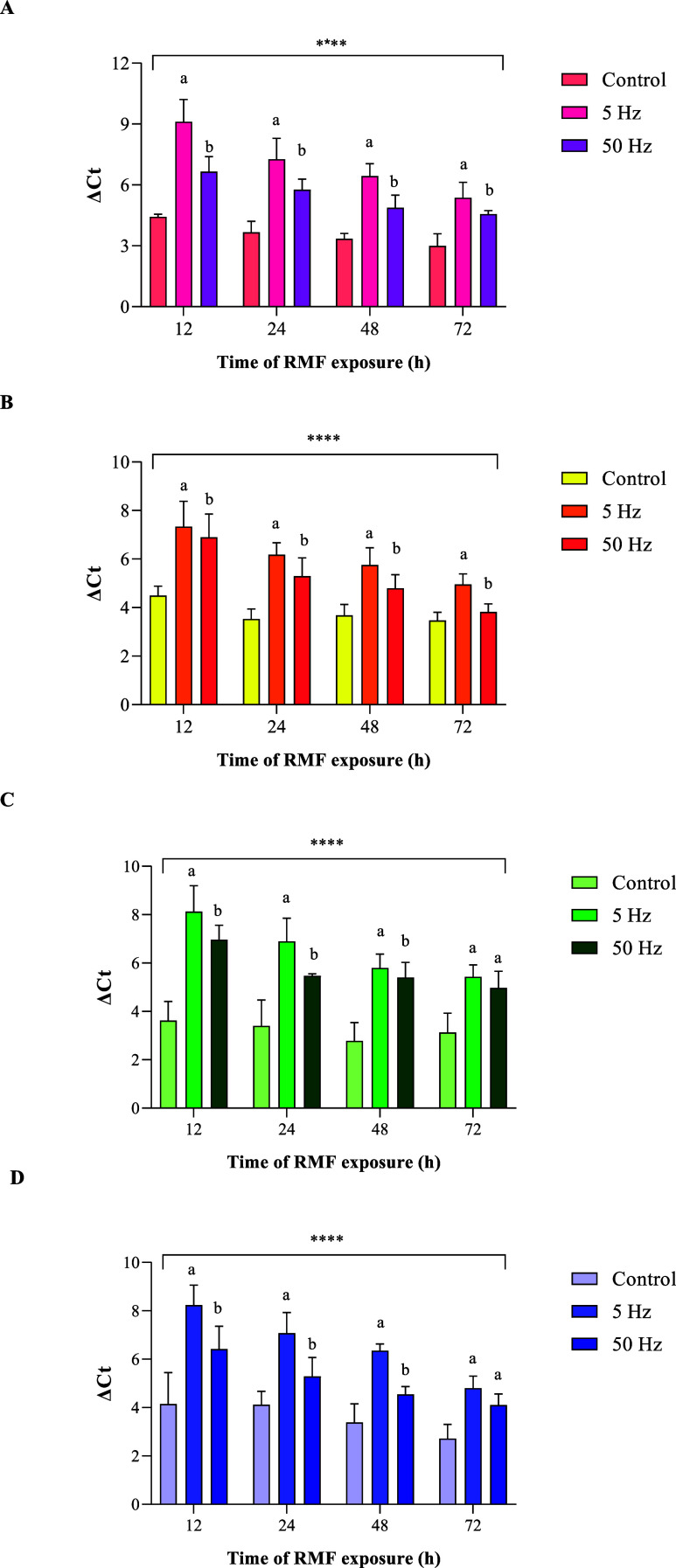
Relative expression levels of genes (a) *bcsA,* (b) *bcsB,* (c) *bcsC*, and (d) *bcsD* in *K. xylinus* cells exposed to the RMF at frequencies of 5 and 50 Hz and unexposed controls during 72 h of incubation.

Notably, the most substantial changes in *bcs* gene expression were observed at the lower RMF applied (5 Hz). It is widely acknowledged that the effect of MFs on bacterial cells is primarily influenced by frequency, as this is the main factor determining the physical characteristics of the magnetic signal.^36,37^ Simulative calculations presented in our previous study^38^ demonstrated that at 5 Hz, the amplitude of the RMF is characterized by a ten time-longer period between states of maximum magnetic induction strength than the RMF of 50 Hz (100 vs 10 ms, respectively). At a frequency of 5 Hz, the expression of the *bcs*A and *bcs*B genes at all time points was higher compared with the results obtained at 50 Hz. For the *bcs*C and *bcs*D genes, the expression levels observed at 5 and 50 Hz were statistically indistinguishable only after 72 h of exposure. At all other time points, significant differences in expression were observed between these two frequencies. It is well known that changes in gene expression can be induced when microorganisms are exposed to naturally occurring stress factors such as heat shock and ultraviolet (UV) irradiation. Furthermore, it can be assumed that exposure to MFs may serve as an additional external stressor for bacteria.^15,21^ In this context, the increased expression of *bcs* genes in cells exposed to the RMF is particularly understandable, as BC is secreted to protect bacteria from UV radiation and other external environmental factors.^39^

The “*” symbol indicates statistically significant differences between RMF-exposed BC and unexposed control BC. * *p* < 0.05; ** *p* < 0.01; ***p < 0.001; **** *p* < 0.0001. Different letters (a, b) indicate statistically significant differences between samples exposed to the RMF of different frequencies (Hz) within a specific time point.

Previous studies have demonstrated that MFs can interfere with electron transport chains, disrupt redox balance, and alter membrane potential, ultimately leading to increased ROS generation in both prokaryotic and eukaryotic systems.^40–42^ Furthermore, in various bacterial species, ROS not only act as damaging agents but also function as signaling molecules that activate stress responses and modulate gene networks involved in biofilm formation and exopolysaccharide synthesis.^43,44^ For instance, exposure of *E. coli* cultures to a 60 Hz MF enhanced the biosynthesis of σ∧32, a transcription factor that regulates stress response promoters.^45^ In bacterial conjugation experiments using an *E. coli* strain carrying the transposable element Tn5, MF exposure stimulated transposition activity, which was mediated by the synthesis and accumulation of heat shock proteins *DnaK* and *DnaJ*.^46^ Moreover, MF exposure has been shown to significantly elevate dehydrogenase activity and intracellular ATP levels, both key indicators of bacterial stress responses.^8^ Additionally, stress-related genes such as *relA* and *spoT,* which regulate the levels of guanosine tetra- and pentaphosphate (alarmones involved in the stringent response), along with *dnaK* and *groEL*, which are involved in protein folding, were upregulated in *Enterococcus* and *Salmonella* species following MF exposure.^16^ These findings support a prevailing hypothesis that MFs interact with biological systems primarily through the generation of ROS, including superoxide anion (O_2_^–^), singlet oxygen (^1^O_2_), hydrogen peroxide (H_2_O_2_), and hydroxyl radicals (•OH). These ROS can function as signaling molecules, initiating transduction cascades and reacting with various biomolecules.^47,48^

Analysis of *bcs* gene expression revealed a temporal pattern, with expression levels decreasing as the incubation time increased. The most pronounced upregulation of *bcs* genes was observed in *K. xylinus* cells after 12 h of exposure to the RMF. At this time point, cells exposed to a 5 Hz RMF showed an average increase in *bcs* gene expression of 74% compared with unexposed controls, while those exposed to a 50 Hz RMF exhibited a 32% increase. Both of these changes were statistically significant (*p* < 0.001). This observation suggests that the impact of the RMF on *bcs* gene expression is most potent during the initial stages of exposure, with the effect diminishing over prolonged incubation periods. It can also be assumed that the observed differences in *bcs* gene expression may be attributed to the sensitivity of *K. xylinus* cells in planktonic versus biofilm forms.

It is important to contextualize these findings within the process of BC biosynthesis by *K. xylinus*. Initially, *K xylinus* cells exist in a planktonic state. In the early production stage, cells begin to secrete extracellular cellulose fibrils, which gradually form island-like fragments on the medium surface (Figure S7). As cultivation continues, these fragments coalesce and thicken, forming a cellulose membrane. The thickening membrane progressively impedes the diffusion of atmospheric oxygen into the medium. By 48 h of cultivation, most, if not all, planktonic cells become embedded within the cellulose matrix. This cellulose pellicle, comprising cellulose fibrils and bacterial cells, is often classified as a floating biofilm.^49^ This progression from planktonic cells to a biofilm state is crucial for understanding the temporal changes in gene expression and cellular behavior observed in *K. xylinus* cultures. Considering the two distinct forms of *K. xylinus* that dominate at different stages of the fermentation process (planktonic cells initially, transitioning to an embedded biofilm state over time), it is reasonable to assume that the response of the bacterial cells to RMF exposure may also vary across these developmental stages. Furthermore, it is important to note that bacteria embedded within biofilms generally exhibit increased resistance to stress factors compared to their planktonic counterparts.^50^ Therefore, it can be suggested that the higher expression of *bcs* genes observed during the initial 12 h of incubation (in comparison to subsequent time points) is attributable to the increased sensitivity of *K. xylinus* cells in their planktonic form to RMF exposure, as opposed to the cells that have become immobilized within the developing cellulose matrix.

Over the past few years, numerous studies have outlined various strategies to enhance the expression of *bcs* genes, with the aim of improving the productivity of BC synthesis. According to Morgan et al.,^51^ both *bcs*A and *bcs*B must be upregulated to increase BC biosynthesis, as functional BcsA is stabilized by its interaction with the BcsB regulatory subunit in the periplasm. Mangayil et al.^52^ further indicated that while both *bcs*A and *bcs*B are directly involved in the BC synthesis process, overexpression of *bcs*A alone does not lead to a corresponding increase in BC yield. Furthermore, Augimeri and Strap^8^ reported that the phytohormone ethylene directly enhances BC biosynthesis by upregulating the expression of *bcs*A and *bcs*B, without any corresponding increase in the expression of *bcs*C and *bcs*D. However, several models proposed by Brown and Saxena^53^ suggest that the processes of assembly and secretion of BC, regulated by *bcsD* and *bcsC*, respectively, are likely rate-limiting steps in its synthesis. Yang et al.^54^ reported an increase in BC yield through the overexpression of only *bcsC* and *bcsD*, using a modified culture medium characterized by lower viscosity. Nojima et al.^55^ showed that the deletion of *bcs*C, similar to the deletion of *bcsA* and *bcsB*, completely inhibits the BC production process. Yang et al.^54^ further demonstrated that overexpression of the *bcsD* gene enhances the crystallinity of BC, the degree of polymerization (DP), tensile strength, and elongation at break, potentially due to the formation of a denser network of BC microstructures, as observed through scanning electron microscopy (SEM). According to Mehta et al.,^56^ in the absence of *bcsD*, predominantly short, amorphous type II cellulose is produced, leading to a 90% decrease in BC production. It can be concluded that a significant increase in BC production efficiency is dependent on the coordinated overexpression of all of the *bcs* genes. Additionally, the overexpression of *bcs*D, in particular, has been shown to influence the quality properties of generated BC.

In this context, the enhanced efficiency of BC production observed in the present study may be influenced not only by the upregulation of *bcs* genes but also by additional physicochemical factors, particularly interactions between the RMF and electrically charged molecules present in the culture medium and within the cell cytoplasm. These findings underscore the complex, multifactorial nature of optimizing BC biosynthesis, which requires an integrated understanding of both genetic regulation and environmental conditions.^25,28^ The culture medium contains a variety of electrolytes, including Na^+^, K^+^, Mg^2+^, and NH_4_^+^, along with the corresponding anions such as sulfate, phosphate, and chloride. In parallel, microbial cells comprise ionic solutions, proteins, and lipids—components that are susceptible to the influence of magnetic and induced electric fields.^57,58^ The interaction between the RMF and these charged species may promote microscale fluid motion (“micromixing”) within the medium, thereby enhancing mass transfer at the cell–liquid interface and modulating transport mechanisms across cellular membranes.^57,58^

It is also noteworthy that only a subset of the total number of *K. xylinus* cells immobilized within the aerobic zone of the cellulose membrane is actively engaged in BC production. Cells located in the deeper, more anoxic zones of the membrane remain relatively inactive and do not contribute significantly to cellulose synthesis. In this context, it can be suggested that the application of RMFs may offer additional benefits beyond just the upregulation of the *bcs* gene expression. The vibration, localized mixing of the bioliquids, and the loose, nanostructured nature of the BC matrix itself may serve to stimulate the metabolism of *K. xylinus* cells residing in the lower, less aerobic layers of the cellulose membrane. This increased metabolic activity in the deeper zones of the biofilm could be another factor positively influencing the overall biosynthesis process of this valuable biomaterial. By leveraging the multifaceted impacts of the RMF on both genetic regulation and biophysical properties of the culture system, we may be able to further optimize the efficiency and yield of bacterial cellulose production. This holistic understanding of the underlying mechanisms is crucial for developing effective strategies to enhance the biotechnological process.

## Conclusions

The results presented in this study demonstrated that the exposure of *K. xylinus* cells to an RMF resulted in a significant increase in BC production efficiency. This enhanced BC yield was directly linked to the upregulated expression of the *bcs* (*bcsABCD*) genes, which encode the key enzymes involved in cellulose biosynthesis. Statistically significant increases in *bcs* gene expression were observed in *K. xylinus* cells exposed to the RMF at both 5 and 50 Hz frequencies. However, the most substantial changes in *bcs* gene expression were detected at a lower RMF frequency of 5 Hz. Furthermore, the study revealed that the increased sensitivity of *K. xylinus* cells in their planktonic form to RMF exposure, as opposed to cells immobilized within the developing bacterial cellulose matrix, was a key factor contributing to the observed temporal patterns in *bcs* gene expression. In conclusion, these findings demonstrate that the RMF has the potential to be utilized as a tool to modulate gene expression in bacteria with significant biotechnological potential, such as those that produce high-value biopolymers such as BC. The ability to fine-tune genetic regulation through physical stimuli provides new opportunities for optimizing the productivity and efficiency of these microbial systems.

## Supplementary Material



## Notes

Additional experimental details, including parameters of the RMF generator, stability analyses of the primers used in the experiment, and the results regarding the biosynthesis and assessment of the physicochemical properties of bacterial cellulose (FTIR and SEM) (PDF)

## References

[1] Distler T., Huemer K., Leitner V., Bischof R. H., Groiss H., Guebitz G. M. (2023). Production of Bacterial Cellulose by *Komagataeibacter
Intermedius* from Spent Sulfite Liquor. Bioresour. Technol. Rep..

[2] Singhania R. R., Patel A. K., Tsai M. L., Chen C. W., Di Dong C. (2021). Genetic Modification
for Enhancing Bacterial Cellulose Production and its Applications. Bioengineering.

[3] Picheth G., Pirich C. L., Sierakowski M. R., Woehl M. A., Sakakibara C. N., de Souza C. F., Martin A. A., da Silva R., de Freitas R. A. (2017). Bacterial
Cellulose in Biomedical Applications: A Review. Int. J. Biol. Macromol..

[4] Horue M., Silva J. M., Berti I. R., Brandão L. R., Barud H. D. S., Castro G. R. (2023). Bacterial Cellulose-Based
Materials
as Dressings for Wound Healing. Pharmaceutics.

[5] Choi S. M., Rao K. M., Zo S. M., Shin E. J., Han S. S. (2022). Bacterial
Cellulose and its Applications. Polymers.

[6] Infante-Neta A. A., D’Almeida A. P., Albuquerque T. L. (2024). Bacterial Cellulose in Food Packaging:
A Bibliometric Analysis and Review of Sustainable Innovations and
Prospects. Processes.

[7] Żywicka A., Peitler D., Rakoczy R., Junka A. F., Fijałkowski K. (2016). Wet and Dry
Forms of Bacterial Cellulose Synthetized by Different Strains of *Gluconacetobacter xylinus* as Carriers for Yeast Immobilization. Appl. Biochem. Biotechnol..

[8] Augimeri R. V., Strap J. L. (2015). The Phytohormone
Ethylene Enhances Cellulose Production,
Regulates CRP/FNRKX Transcription and Causes Differential Gene Expression
Within the Bacterial Cellulose Synthesis Operon of *Komagataeibacter* (*Gluconacetobacter*) *xylinus* ATCC
53582. Front. Microbiol..

[9] Al-Janabi S. S., Shawky H., El-Waseif A. A., Farrag A. A., Abdelghany T. M., El-Ghwas D. E. (2022). Novel Approach of
Amplification and Cloning of *Bacterial Cellulose Synthesis
(BCS)* Operon From *Gluconoacetobacter hansenii*. Gene
Rep..

[10] Bimmer M., Mientus M., Klingl A., Ehrenreich A., Liebl W. (2022). The Roles of the Various Cellulose
Biosynthesis Operons in *Komagataeibacter hansenii* ATCC 23769. Appl. Environ. Microbiol..

[11] Kondo T., Nakamura Y., Nojima S., Yao M., Imai T. (2022). The *BcsD* Subunit of Type I Bacterial Cellulose Synthase Interacts
Dynamically with the *BcsAB* Catalytic Core Complex. FEBS Lett..

[12] Jacek P., Dourado F., Gama M., Bielecki S. (2019). Molecular Aspects of
Bacterial Nanocellulose Biosynthesis. Microb.
Biotechnol..

[13] Mousavian-Roshanzamir S., Makhdoumi-Kakhki A. (2017). The Inhibitory Effects of Static Magnetic Field on
Escherichia coli from Two Different Sources at Short Exposure Time. Rep. Biochem. Mol. Biol..

[14] Bayır E., Bilgi E., Şendemir-Ürkmez A., HameŞ-KocabaŞ E. (2015). The Effects Of Different Intensities,
Frequencies and Exposure Times of Extremely Low-Frequency Electromagnetic
Fields on The Growth of *Staphylococcus aureus* and
Escherichia coli O157: H7. Electromagn. Biol.
Med..

[15] Li S. H., Chow K. C. (2001). Magnetic
Field Exposure Induces DNA Degradation. Biochem.
Biophys. Res. Commun..

[16] El
May A., Snoussi S., Ben Miloud N., Maatouk I., Abdelmelek H., Ben Aïssa R., Landoulsi A. (2009). Effects of Static Magnetic Field
on Cell Growth, Viability, and Differential Gene Expression in Salmonella. Foodborne Pathog. Dis..

[17] El
May A., Zouaoui J., Snoussi S., Ben Mouhoub R., Landoulsi A. (2021). *RelA* and *SpoT* Gene
Expression is Modulated in Salmonella Grown Under Static Magnetic
Field. Curr. Microbiol..

[18] Mouhoub R. B., Mansouri A., Aliliche K., Beghalem H., Landoulsi A., El May A. (2017). Unraveling the Expression
of Genes Involved in the
Biosynthesis Pathway of Cardiolipin and Phosphatidylethanolamine in
Salmonella Hadar Grown Under Static Magnetic Field 200 mT. Microb. Pathog.

[19] Kwiatkowski P., Tabiś A., Fijałkowski K., Masiuk H., Łopusiewicz Ł., Pruss A., Sienkiewicz M., Wardach M., Kurzawski M., Guenther S., Bania J., Dołęgowska B., Wojciechowska-Koszko I. (2022). Regulatory and Enterotoxin Gene Expression And Enterotoxins
Production in *Staphylococcus aureus* FRI913 Cultures
Exposed to a Rotating Magnetic Field and *Trans*-Anethole. Int. J. Mol. Sci..

[20] Fijałkowski K., Peitler D., Żywicka A., Rakoczy R. (2016). The Effect of Rotating
Magnetic Field on Enterotoxins Genes Expression in *Staphylococcus
aureus* Strains. J. Magn..

[21] Potenza L., Cucchiarini L., Piatti E., Angelini U., Dachà M. (2004). Effects of
High Static Magnetic Field Exposure on Different DNAs. Bioelectromagnetics.

[22] Fijałkowski K., Żywicka A., Drozd R., Niemczyk A., Junka A. F., Peitler D., Kordas M., Konopacki M., Szymczyk P., El Fray M. (2015). Modification of Bacterial
Cellulose Through Exposure to the Rotating Magnetic field. Carbohydr. Polym..

[23] Fijałkowski K., Rakoczy R., Żywicka A., Drozd R., Zielińska B., Wenelska K., Cendrowski K., Peitler D., Kordas M., Konopacki M. (2016). Time Dependent Influence of Rotating Magnetic
Field on Bacterial Cellulose. Int. J. Polym.
Sci..

[24] Fijałkowski K., Żywicka A., Drozd R., Junka A. F., Peitler D., Kordas M., Konopacki M., Szyczyk P., El Fray M., Rakoczy R. (2016). Increased Yield and
Selected Properties of Bacterial
Cellulose Exposed to Different Modes of a Rotating Magnetic Field. Eng. Life Sci..

[25] Drozd R., Charęza M., Żywicka A., Kowalska U., Rakoczy R., Kordas M., Konopacki M., Junka A. F., Fijałkowski K. (2021). Exposure to
Non-Continuous Rotating Magnetic Field Induces Metabolic Strain-Specific
Response of *Komagataeibacter xylinus*. Biochem. Eng. J..

[26] Żywicka A., Ciecholewska-Juśko D., Drozd R., Rakoczy R., Konopacki M., Kordas M., Junka A. F., Migdał P., Fijałkowski K. (2021). Preparation of *Komagataeibacter xylinus* Inoculum for Bacterial Cellulose Biosynthesis Using Magnetically
Assisted External-Loop Airlift Bioreactor. Polymers.

[27] Ciecholewska-Juśko D., Broda M., Żywicka A., Styburski D., Sobolewski P., Gorący K., Migdał P., Junka A., Fijałkowski K. (2021). Potato Juice,
a Starch Industry Waste,
as a Cost-Effective Medium for the Biosynthesis of Bacterial Cellulose. Int. J. Mol. Sci..

[28] Fijałkowski K., Żywicka A., Drozd R., Junka A. F., Peitler D., Kordas M., Konopacki M., Szymczyk P., Rakoczy R. (2017). Increased
water content in bacterial cellulose synthesized under rotating magnetic
fields. Electromagn. Biol. Med..

[29] Vandesompele J., De Preter K., Pattyn F., Poppe B., Van Roy N., De Paepe A., Speleman F. (2002). Accurate Normalization of Real-Time
Quantitative RT-PCR Data by Geometric Averaging of Multiple Internal
Control Genes. Genome Biol..

[30] Abdelraof M., Hasanin M. S., Farag M. M., Ahmed H. Y. (2019). Green Synthesis
of Bacterial Cellulose/Bioactive Glass Nanocomposites: Effect of Glass
Nanoparticles on Cellulose Yield, Biocompatibility and Antimicrobial
Activity. Int. J. Biol. Macromol..

[31] Klumpp S., Hwa T. (2014). Bacterial Growth: Global
Effects on Gene Expression, Growth Feedback
and Proteome Partition. Curr. Opin. Biotechnol..

[32] Sampaio N. M. V., Blassick C. M., Andreani V., Lugagne J. B., Dunlop M. J. (2022). Dynamic
Gene Expression and Growth Underlie Cell-To-Cell Heterogeneity in
Escherichia coli Stress Response. Proc. Natl.
Acad. Sci. U. S. A..

[33] Fijałkowski K., Nawrotek P., Struk M., Kordas M., Rakoczy R. (2013). The Effects
of Rotating Magnetic Field on Growth Rate, Cell Metabolic Activity
and Biofilm Formation by *Staphylococcus aureus* and *Escherichia coil*. J. Magn..

[34] Fijałkowski K., Nawrotek P., Struk M., Kordas M., Rakoczy R. (2015). Effects of
Rotating Magnetic Field Exposure on the Functional Parameters of Different
Species of Bacteria. Electromagn. Biol. Med..

[35] Nawrotek P., Fijałkowski K., Struk M., Kordas M., Rakoczy R. (2014). Effects of
50 Hz Rotating Magnetic Field on the Viability of Escherichia
coli and *Staphylococcus aureus*. Electromagn. Biol. Med..

[36] Fojt L., Strasák L., Vetterl V., Smarda J. (2004). Comparison of the Low-Frequency
Magnetic Field Effects on Bacteria Escherichia coli, *Leclercia
adecarboxylata* and *Staphylococcus aureus*. Bioelectrochemistry.

[37] Ahmed I., Istivan T., Cosic I., Pirogova E. (2013). Evaluation of the Effects
of Extremely Low Frequency (ELF) Pulsed Electromagnetic Fields (PEMF)
On Survival of the Bacterium *Staphylococcus aureus*. EPJ. Nonlinear Biomed. Phys..

[38] Woroszyło M., Ciecholewska-Juśko D., Junka A., Drozd R., Wardach M., Migdał P., Szymczyk-Ziółkowska P., Styburski D., Fijałkowski K. (2021). Rotating Magnetic Field Increases
β-Lactam Antibiotic Susceptibility of Methicillin-Resistant *Staphylococcus aureus* Strains. Int.
J. Mol. Sci..

[39] Mauro F., Corrado B., De Gregorio V., Lagreca E., Di Natale C., Vecchione R., Netti P. A. (2024). Exploring the Evolution of Bacterial
Cellulose Precursors and Their Potential Use as Cellulose-Based Building
Blocks. Sci. Rep..

[40] Shimizu N., Hosogi N., Hyon G. S. (2006). Reactive
oxygen species
(ROS) Generation and ROS-Induced Lipid Peroxidation are Associated
with Plasma Membrane Modifications in Host Cells in Response to AK-Toxin
I From *Alternaria alternata* Japanese Pear Pathotype. J. Gen. Plant. Pathol..

[41] Wang H., Zhang X. (2017). Magnetic Fields and Reactive Oxygen
Species. Int. J. Mol. Sci..

[42] Zhao X., Hong Y., Drlica K. (2015). Moving Forward
with reactive oxygen
species involvement in antimicrobial lethality. J. Antimicrob. Chemother..

[43] Maksimova Y., Zorina A., Nesterova L. (2023). Oxidative
Stress Response and E.
coli Biofilm Formation under the Effect of Pristine and Modified Carbon
Nanotubes. Microorganisms.

[44] Chua S. L., Ding Y., Liu Y., Cai Z., Zhou J., Swarup S., Drautz-Moses D. I., Schuster S. C., Kjelleberg S., Givskov M., Yang L. (2016). Reactive Oxygen
Species Drive Evolution
of Pro-Biofilm Variants in Pathogens by Modulating Cyclic-di-GMP Levels. Open Biol..

[45] Chow K. C., Tung W. L. (2000). Magnetic Field Exposure Stimulates Transposition Through
the Induction of DNA K/J Synthesis. Biochem.
Biophys. Res. Commun..

[46] Chow K. C., Tung W. L. (2000). Magnetic Field Exposure Enhances DNA Repair Through
The Induction of DNA K/J Synthesis. FEBS Lett..

[47] Okano H. (2008). Effects of
Static Magnetic Fields in Biology: Role of Free Radicals. Front Biosci..

[48] Mundell J.
W., Brier M. I., Orloff E., Stanley S. A., Dordick J. S. (2024). Alternating
Magnetic Fields Drive Stimulation of Gene Expression Via Generation
of Reactive Oxygen Species. iScience.

[49] Gilmour K. A., Aljannat M., Markwell C., James P., Scott J., Jiang Y., Torun H., Dade-Robertson M., Zhang M. (2023). Biofilm Inspired Fabrication of Functional
Bacterial Cellulose Through *Ex-Situ* and *In-Situ* Approaches. Carbohydr. Polym..

[50] Wicaksono W. A., Erschen S., Krause R., Müller H., Cernava T., Berg G. (2022). Enhanced Survival of
Multi-Species
Biofilms Under Stress is Promoted by Low-Abundant but Antimicrobial-Resistant
Keystone Species. J. Hazard. Mater..

[51] Morgan J. L., McNamara J. T. W., Zimmer J. (2014). Mechanism
of Activation of Bacterial
Cellulose Synthase by Cyclic di-GMP. Nat. Struct.
Mol. Biol..

[52] Mangayil R., Rajala S., Pammo A., Sarlin E., Luo J., Santala V., Karp M., Tuukkanen S. (2017). Engineering
and Characterization of Bacterial Nanocellulose Films as Low Cost
and Flexible Sensor Material. ACS Appl. Mater.
Interfaces.

[53] Brown R. M., Saxena I. M. (2000). Cellulose Biosynthesis: A Model for
Understanding the Assembly of Biopolymers. Plant
Physiol. Biochem..

[54] Yang L., Zhu X., Chen Y., Wang J. (2024). Enhanced Bacterial
Cellulose Production
In *Gluconacetobacter xylinus* by Overexpression ff
Two Genes (*bscC* and *bcsD*) and a
Modified Static Culture. Int. J. Biol. Macromol..

[55] Nojima S., Fujishima A., Kato K., Ohuchi K., Shimizu N., Yonezawa K., Tajima K., Yao M. (2017). Crystal Structure of
the Flexible Tandem Repeat Domain of Bacterial Cellulose Synthesis
Subunit C. Sci. Rep..

[56] Mehta K., Pfeffer S., Brown R. M. (2015). Characterization
of an *acsD* Disruption Mutant Provides Additional
Evidence for the Hierarchical
Cell-Directed Self-Assembly of Cellulose in *Gluconacetobacter
xylinus*. Cellulose.

[57] Anton–leberre V., Haanappel E., Marsaud N., Trouilh L., Benbadis L., Boucherie H., Massou S., Francois J. M. (2010). Exposure to High
Static of Pulsed Magnetic Fields Does Not Affect Cellular Processes
in the Yeast *Saccharomyces cerevisiae*. Bioelectromagnetics.

[58] Gaafar E. S., Hanafy M. S., Tohamy E. Y. (2008). The Effect
of Electromagnetic Field
on Protein Molecular Structure of E. coli and Its Pathogenesis. Rom. J. Biophys..

