# Supplementary material for: Increased Expression of Bacterial Cellulose Synthase Genes in Komagataeibacter Xylinus Exposed to a Rotating Magnetic Field
Source: Biomacromolecules. 2025 Aug 29;26(10):6542–51. doi: 10.1021/acs.biomac.5c00653 (PMC12522092; doi:10.1021/acs.biomac.5c00653)
Supplement: Supplementary file 1 [file bm5c00653_si_001.pdf]

Increased expression of bacterial cellulose synthase genes in *Komagataeibacter xylinus* exposed to rotating magnetic field

Anna Żywicka[1], Aleksandra Dunislawska[2], Karol Fijałkowski[1]*

[1] Department of Microbiology and Biotechnology, West Pomeranian University of Technology in Szczecin, Piastow 45, 70-311 Szczecin, Poland; anna.zywicka@zut.edu.pl, karol.fijalkowski@zut.edu.pl

[2] Department of Animal Biotechnology and Genetics, Bydgoszcz University of Science and Technology, Mazowiecka 28, Bydgoszcz 85-084, Poland; aleksandra.dunislawska@pbs.edu.pl

Supplementary tables captions

Table S1. Magnetic induction values [mT] measured inside the RMF generator at the location of the Falcon tube, corresponding to different applied AC frequencies.

Table S2. The main data of the RMF generator.

Table S3. Sequences and characteristics of primers used for reference gene analysis in this study.

Supplementary figure captions

Fig. S1. Spatial distribution of magnetic field a) in cross-section (h = 10 cm) of RMF-bioreactor in center of representative test tube placed inside RMF-bioreactor.

Figure S2. Stability analysis of candidate reference genes using the RefFinder tool. (https://www.ciidirsinaloa.com.mx/RefFinder-master) (Xie, F.; Xiao, P.; Chen, D.; Xu, L.; Zhang, B. miRDeepFinder: a miRNA Analysis Tool for Deep Sequencing of Plant Small RNAs. *Plant Mol. Biol.* **2012,** *80 (1),* 75-84).

Figure S3. Melting curves and melting peaks for all primer set.

Figure S4. Agarose gel electrophoresis (2%) of PCR products.

Figure S5. FTIR-ATR spectra of unmodified and RMF modified BC.

Figure S6. SEM images of BC: control (C) and RMF-exposed samples.

Figure S7. The process of cellulose membrane formation during 72 h of exposure to RMF.

Supplementary materials

Materials and methods

Table S1. Magnetic induction values [mT] measured inside the RMF generator at the location of the Falcon tube, corresponding to different applied AC frequencies.

	**5 Hz**	**50 Hz**
**MIN**	17.23	17.95
**MAX**	21.88	22.77
**Average**	19.20	19.99

Table S2. The main data of the RMF generator.

No.	Parameter	Description	Value
1.	R_{so}	Stator’s outer radius	110.0 mm
2.	R_{si}	Stator’s inner radius	80.0 mm
3.	l	RMF generator’s length	199.0 mm
4.	p	Number of pole pairs	2
5.	k_s	Number of stator windings in slot	50
6.	s	Number of slots	36
7.	U_n	Nominal phase voltage	230 V
8.	I_n	Nominal phase current	17.1 A
9.	p_b	Number of winding parallel branches	2
10.	R_{ph}	Phase resistance	1.94 Ω
11.	L_{ph}	Phase inductance	8.04 mH

RMF generator is powered by an inverter with the use of U/f = const control. The stator used was from a mass-produced 15 kW four-pole induction motor (INDUKTA S.A., 2SIE160L4, Poland). Since the stator worked without a rotor (there were test samples in place of the rotor), the nature of its operation, from the point of view of power supply, was similar to that of a three-

phase choke. As a result, the induced voltage (back-EMF) reached a relatively small value, therefore it was necessary to limit the supply voltage to a value of 100 V. The stator used is a three-phase wound and star-connected. After applying power to the stator phase bands, magnetic poles were formed inside the stator, which shifted in time according to the frequency of the supply current. Thus, a system configured in this way generated a rotating magnetic field (RMF) inside the stator.

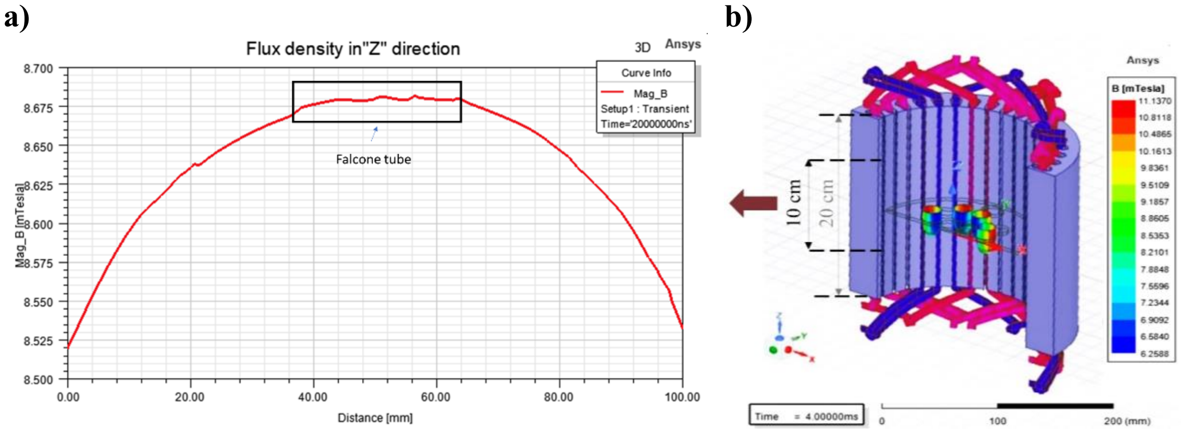

Fig. S1. Spatial distribution of magnetic field a) in cross-section (h = 10 cm) of RMF-bioreactor in center of representative test tube placed inside RMF-bioreactor.

Table S3. Sequences and characteristics of primers used for reference gene analysis in this study.

Primer	Encoded protein	Sequence (5'→3')	Tm (°C)	Amplicon length (bp)
23Sr RNA	23S ribosomal RNA subunit	F: TGAGCTGGGTTTAGAACGTCGTG<br>R: ACACCTGGCCTATTGACGTGATG	62.0	255
gyrA	DNA gyrase subunit A	F: TTGACCGAAATTCCCGAACA<br>R: TCGCGCATGGAATACAGGAT	59.0	176
gyrB	DNA gyrase subunit B	F: TCTCGTCACAGACCAAGGACAAG<br>R: CTTCCTTGGGGTGGGTTTCAAAC	60.0	108
recA	Recombinase A	F: AGGCTCCGGGTATTGACAAA<br>R: ATCACATCGACCTCCGTCTT	59.0	121
rho	transcription termination factor	F: TGCATCTCGCCGAACTCAAG<br>R: GAAGCCGAAGCCATCCGACA	61.0	191

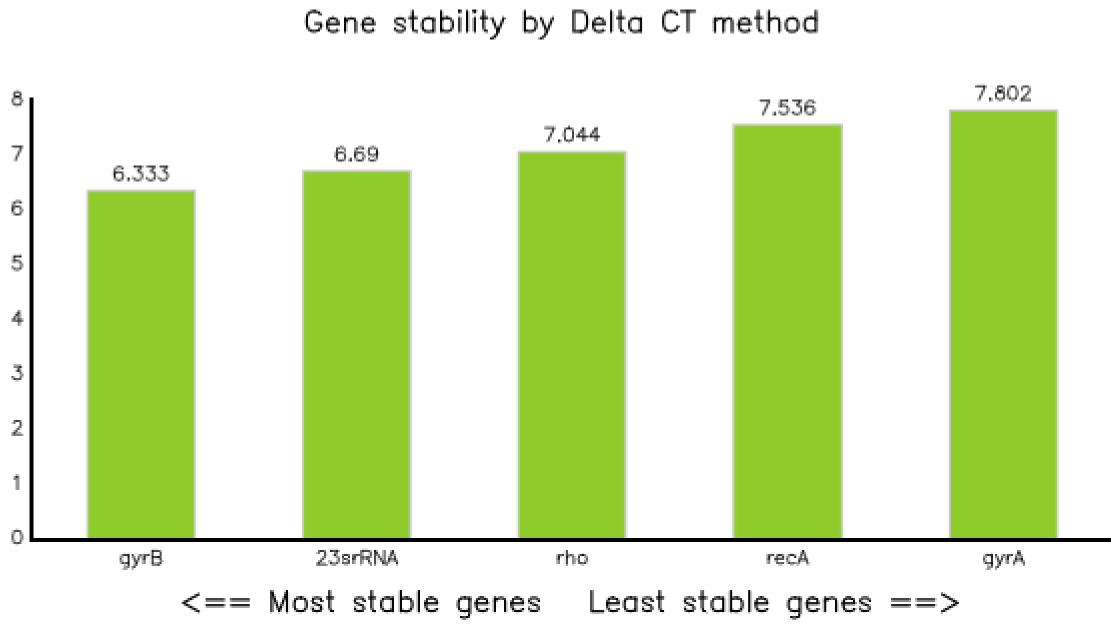

Figure S2. Stability analysis of candidate reference genes using the RefFinder tool. (https://www.ciidirsinaloa.com.mx/RefFinder-master) (Xie, F.; Xiao, P.; Chen, D.; Xu, L.; Zhang, B. miRDeepFinder: a miRNA Analysis Tool for Deep Sequencing of Plant Small RNAs. *Plant Mol. Biol.* **2012,** *80 (1),* 75-84).

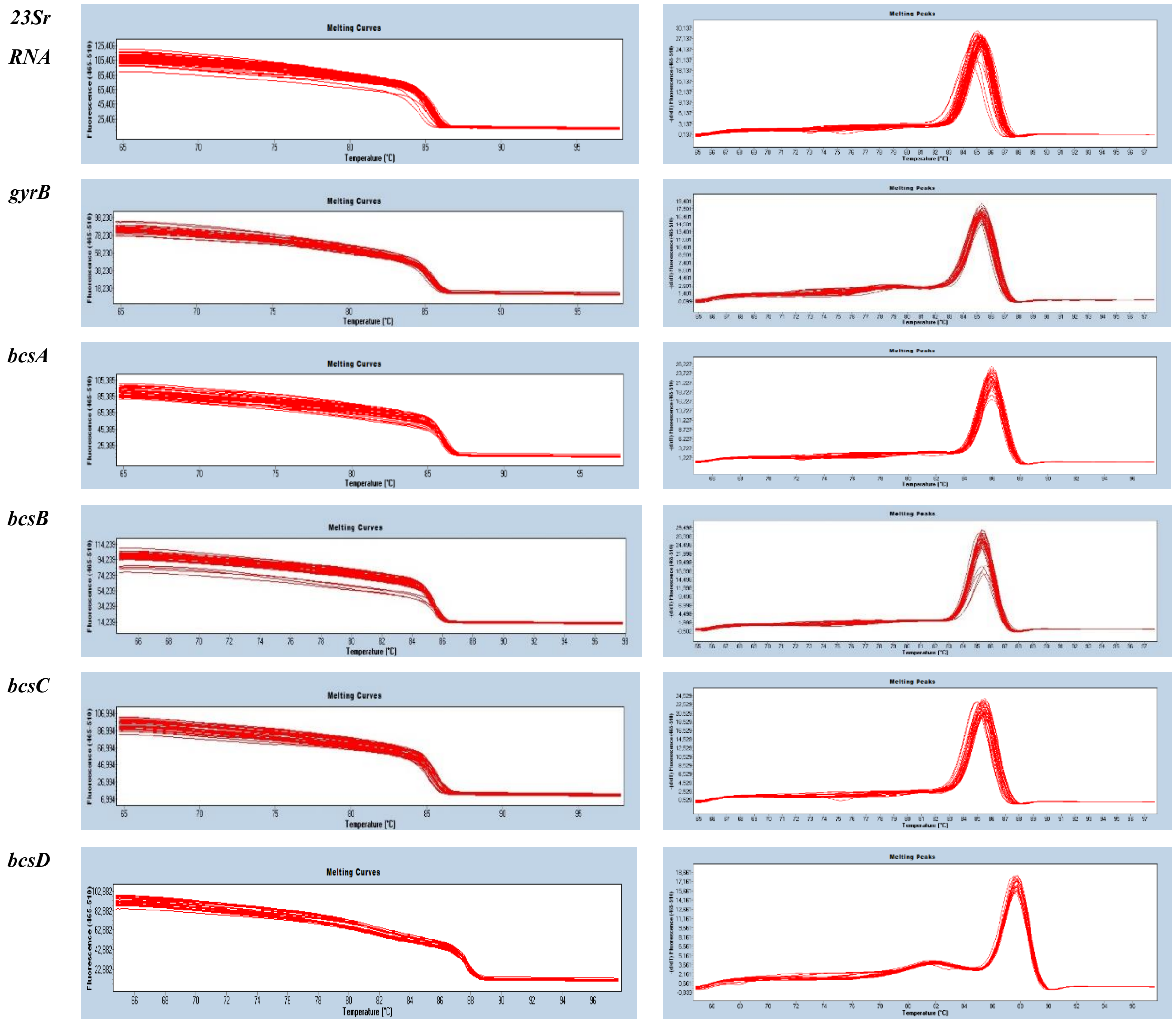

Figure S3. Melting curves and melting peaks for all primer set.

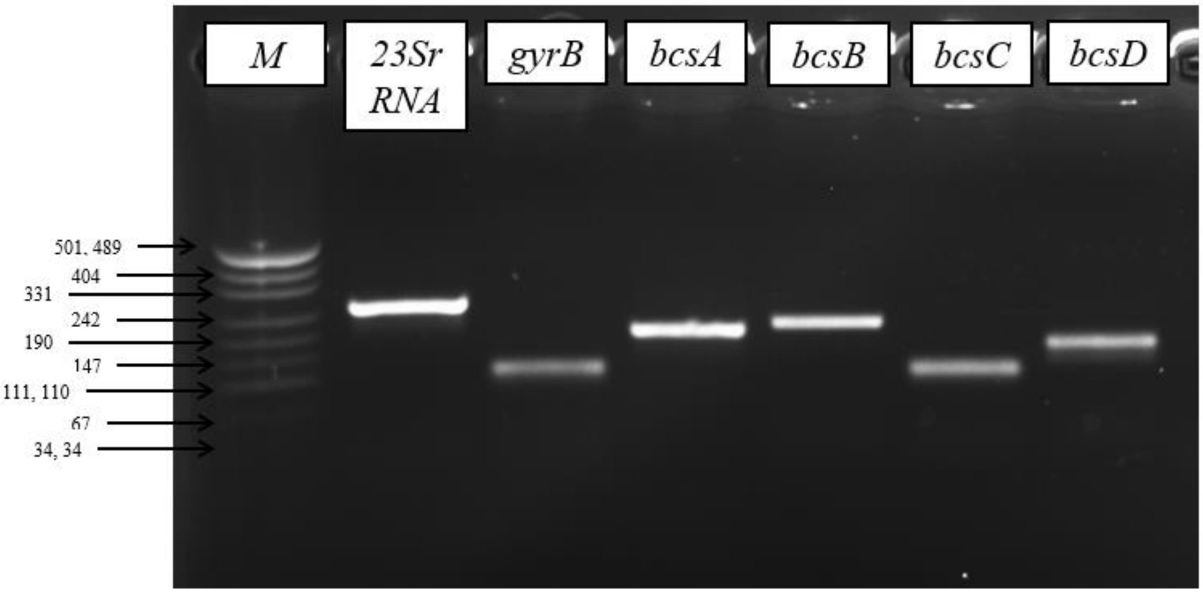

Figure S4. Agarose gel electrophoresis (2%) of PCR products.

Results and discussion

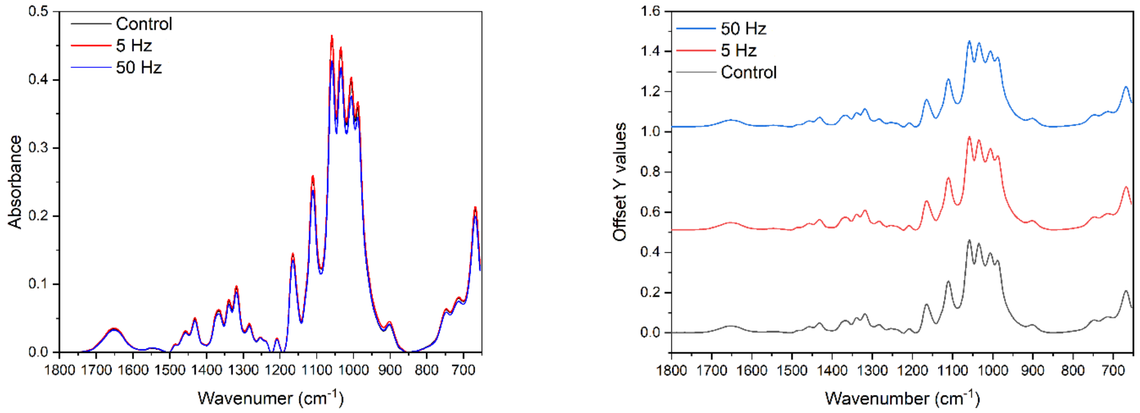

Figure S5. FTIR-ATR spectra of unmodified and RMF modified BC.

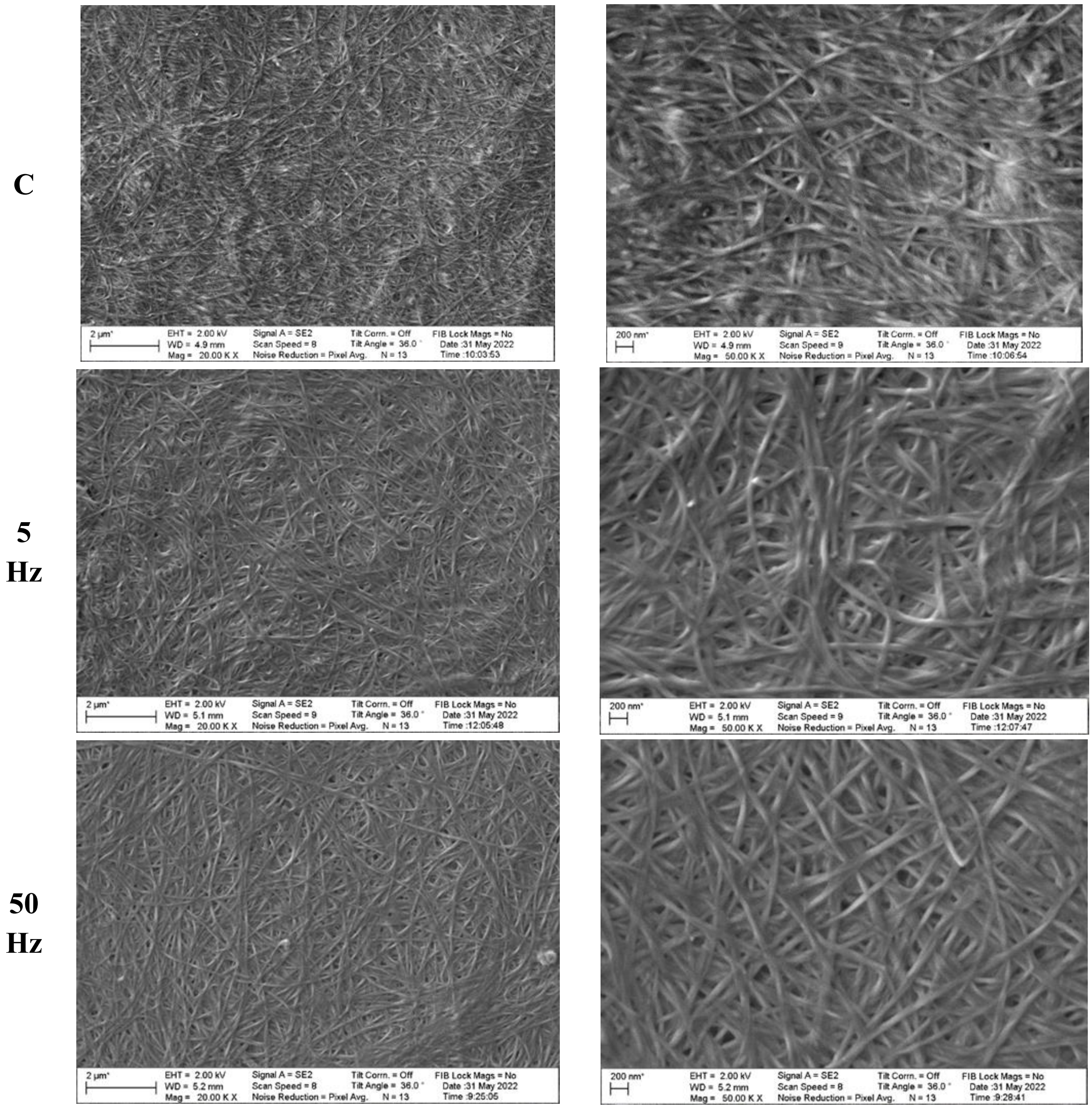

Figure S6. SEM images of BC: control (C) and RMF-exposed samples.

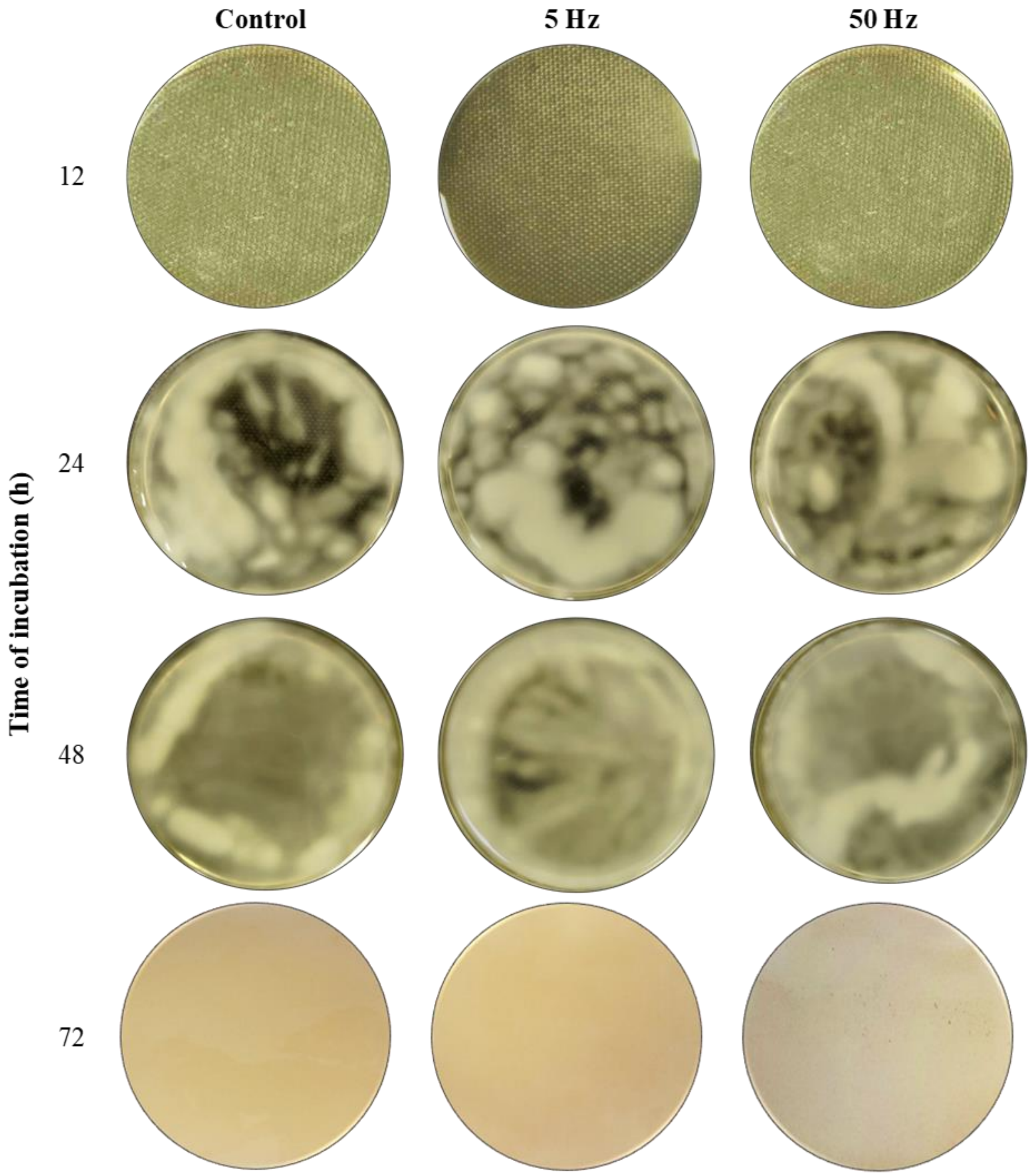

Figure S7. The process of cellulose membrane formation during 72 h of exposure to RMF.
